# Gene Expression Modification by an Autosomal Inversion Associated With Three Male Mating Morphs

**DOI:** 10.3389/fgene.2021.641620

**Published:** 2021-06-04

**Authors:** Jasmine L. Loveland, David B. Lank, Clemens Küpper

**Affiliations:** ^1^Research Group for Behavioural Genetics and Evolutionary Ecology, Max Planck Institute for Ornithology, Seewiesen, Germany; ^2^Department of Biological Sciences, Simon Fraser University, Burnaby, BC, Canada

**Keywords:** chromosomal inversion, alternative reproduction strategies, steroidogenic pathway, *Philomachus pugnax*, SDR42E1, aromatase, HSD17B2, *CENPN*

## Abstract

Chromosomal inversions are structural rearrangements that frequently provide genomic substrate for phenotypic diversity. In the ruff *Philomachus pugnax*, three distinct male reproductive morphs (Independents, Satellites and Faeders) are genetically determined by a 4.5 Mb autosomal inversion. Here we test how this stable inversion polymorphism affects gene expression in males during the lekking season. Gene expression may be altered through disruptions at the breakpoints and the accumulation of mutations due to suppressed recombination. We used quantitative PCR to measure expression of 11 candidate inversion genes across three different tissues (liver, adrenal glands and gonads) and tested for allelic imbalance in four inversion genes across 12 males of all three morphs (8 Independents, 2 Satellites, 2 Faeders). We quantified transcripts of *CENPN*, an essential gene disrupted by the inversion at the proximal breakpoint, at different exons distributed near and across the breakpoint region. Consistent with dosage dependent gene expression for the breakpoint gene *CENPN*, we found that expression in Independents was broadly similar for transcripts segments from inside and outside the inversion regions, whereas for Satellites and Faeders, transcript segments outside of the inversion showed at least twofold higher expression than those spanning over the breakpoint. Within the inversion, observed expression differences for inversion males across all four genes with allele-specific primers were consistent with allelic imbalance. We further analyzed gonadal expression of two inversion genes, *HSD17B2* and *SDR42E1*, along with 12 non-inversion genes related to steroid metabolism and signaling in 25 males (13 Independents, 7 Satellites, 5 Faeders). Although we did not find clear morph differentiation for many individual genes, all three morphs could be separated based on gene expression differences when using linear discriminant analysis (LDA), regardless of genomic location (i.e., inside or outside of the inversion). This was robust to the removal of genes with the highest loadings. Pairwise correlations in the expression of genes showed significant correlations for 9–18 pairs of genes within morphs. However, between morphs, we only found a single association between genes *SDR42E1* and *AROM* for Independents and Satellites. Our results suggest complex and wide-ranging changes in gene expression caused by structural variants.

## Introduction

Chromosomal inversions are genomic rearrangements that occur in animals and plants and are associated with local adaptation and speciation (Kirkpatrick, 2010; Dagilis and Kirkpatrick, 2016; Wellenreuther and Bernatchez, 2018; Faria et al., 2019). Inversion polymorphisms, which can be maintained by balancing selection (Kirkpatrick, 2010; Wellenreuther and Bernatchez, 2018), frequently provide the genetic basis for morphological and behavioral diversity (Fuller et al., 2016; Küpper et al., 2016; Lamichhaney et al., 2016; Lindtke et al., 2017; Huang et al., 2018). Changes in gene expression provide one possible mechanism for chromosomal inversions to increase phenotypic variation (Naseeb et al., 2016; Said et al., 2018). Quantifying gene expression differences between inversion alleles is therefore an important step toward characterizing the molecular mechanisms that lead from genotypic to phenotypic variation.

A signature feature of inversions is that they are subject to suppressed recombination, which leads to high genetic differentiation between inverted and non-inverted alleles (Kirkpatrick, 2010). While sequence divergence in regulatory and coding regions alike, requires time for mutations to appear, the new chromosomal rearrangement has the potential to immediately alter gene expression. Consequences of an inversion thus include the loss of a full transcript if a gene is located at a breakpoint, and the disruption of cis-regulatory elements that control transcription of genes near a breakpoint (Huang et al., 2018; Faria et al., 2019; Yan et al., 2020). For example, approximately half of haeomophilia A cases are explained by the impact of an inversion breakpoint that disrupts a coagulation factor gene (Lakich et al., 1993; Antonarakis et al., 1995; Brinke et al., 1996; Cumming, 2004). Allelic imbalance, a change in expression levels between non-inverted and inverted haplotypes, can also be observed for genes in inversion regions (Sun et al., 2018). This imbalance is caused by mutations in regulatory regions. Inversion alleles may then either become over- or underexpressed in heterozygotes. In white-throated sparrows (*Zonotrichia albicollis*), a systematic study on neural tissue revealed that reduced expression of the inversion allele can lead to dosage compensation with the non-inversion allele becoming more expressed (Sun et al., 2018). As a result, in morphs that are heterozygous for the inversion, many genes may show allelic imbalance although total gene expression of these loci is not necessarily altered in comparison to the ancestral morph.

An inherent difficulty in pin-pointing causal associations between an inversion and its associated traits is that variation in gene expression can be restricted, in any number of combinations, to a specific time of the year, sex, tissue type and life stage, to name a few (Imsland et al., 2012; Fuller et al., 2016; Wang et al., 2017; Newhouse et al., 2019). For example, in the Rose-comb chicken (*Gallus gallus*), the comb phenotype is due to ectopic expression of an inversion gene (*MNR2*) in comb tissue during a narrow window of embryonic development (Imsland et al., 2012; Wang et al., 2017). In addition, low fertility and poor sperm motility Rose-comb traits are associated with truncated transcripts and increased testicular expression of an inversion gene (*CCDC108*), most pronounced in birds homozygous for the R1 inversion allele (Imsland et al., 2012). Interestingly, both *MNR2* and *CCDC108* are located near the distal breakpoint of the inversion (Wang et al., 2017) and their phenotypic consequences, in both R1 and R2 inversion alleles can be traced to exon shuffling near breakpoints (Imsland et al., 2012).

Through suppressed recombination, inversions often form supergenes (Schwander et al., 2014), where certain allele combinations are fixed and evolve together. In some prominent examples of adaptive behavioral diversity, these supergenes have captured multiple loci involved in hormone signaling and metabolism (Merritt et al., 2018, 2020; Horton et al., 2020). Steroid hormones often have widespread pleiotropic effects and modulate gene expression genome-wide across many different tissues (Beato, 1993). In addition, steroids such as testosterone and estrogen are extremely important for determining the sexual differentiation of the brain (Steimer and Hutchison, 1980; Balthazart and Ball, 1995; Court et al., 2020). These two hormones can have long-lasting organizational effects on brain development, which is an effective way to exert an entire morph-specific neural circuit by virtue of the delicate relationship between genetic sex, hormonal environment and genetic background.

**FIGURE 1:**
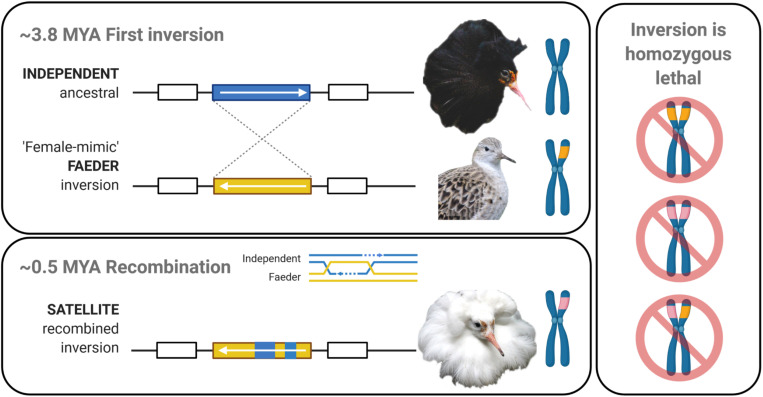
Evolutionary history of the ruff inversion alleles. **Top panel:** Ancestral Independent males have dark ornamental feathers and compete aggressively with other males for matings on leks. The initial inversion (yellow) occurred approximately 3.8 million years ago (MYA) on chromosome 11 (Küpper et al., 2016; Lamichhaney et al., 2016) and led to the “female-mimic” Faeder morph. Faeders are small males that lack ornamental feathers. **Bottom panel:** Recombination between inversion and ancestral alleles is strongly suppressed. Yet, approximately 0.5 MYA, rare recombination events between Faeder and Independent alleles created the Satellite allele (pink). Satellites feature light colored ornamental feathers and display cooperatively with Independent males on leks. **Right panel:** As the inversion is homozygous lethal (Küpper et al., 2016) recombination between inversion alleles does not occur. Created with BioRender.com.

Here we investigate the impact of an autosomal inversion on gene expression in the ruff (*Philomachus pugnax*). This lekking shorebird is characterized by a stable polymorphism that includes three phenotypically and genetically distinct male mating morphs: Independents, Satellites, and Faeders (Lank et al., 2013; Figure 1). Male morph type is determined by a 4.5 Mb inversion located on chromosome 11 that contains no more than 125 genes, with Satellites and Faeders carrying distinct dominant inversion alleles (Küpper et al., 2016; Lamichhaney et al., 2016). Inversion homozygotes are non-viable (Figure 1), thus, all Satellites and Faeders are inversion heterozygotes (i.e., carry one ancestral and one inversion haplotype). During the breeding season, these three morphs showcase alternative reproductive tactics (ARTs) with discrete differences in aggression and courtship displays (Jukema and Piersma, 2006; Küpper et al., 2016). Independent males with ornamental plumage, most often predominantly dark in color, establish courts on leks and are highly aggressive. Independent males form temporary alliances with Satellite males, a morph with lightly colored ornamented feathers, and together they engage in semi-cooperative courtship displays. In contrast, the third morph, female-mimicking Faeder males, do not display any male-typical ornamentation or courtship behaviors (Jukema and Piersma, 2006). Hormonally, inversion morphs consistently have the lowest levels of circulating testosterone, but also higher levels of androstenedione (a testosterone precursor and metabolite), compared to Independent males (Küpper et al., 2016; Loveland et al., 2021). Whether and how higher levels of androstenedione in inversion morphs could produce physiological, morphological or behavioral differences compared to Independents is not known, in part because androstenedione is only a weak activator of the androgen receptor. Given the important role of sex hormones in priming and maintaining seasonal social behavior, the behavioral differences among morphs are presumed to be connected to their respective androgenic differences (Küpper et al., 2016; Loveland et al., 2021). In a recent study, we found that the ability to synthesize testosterone in inversion morphs is severely impaired: stimulation of the hypothalamic–pituitary–gonadal axis with gonadotropin-releasing hormone (GnRH) induced a robust increase in androstenedione, but only a subdued increase in testosterone levels, compared to Independent males (Loveland et al., 2021).

The morphological and behavioral differences between Satellites and Faeders, both inversion carriers, must derive from their particular inversion haplotypes. The Faeder haplotype evolved first 3.8 million years ago (MYA) and the Satellite haplotype appeared just 0.5 MYA, following a rare recombination between the Faeder inversion and one or several Independent alleles (Lamichhaney et al., 2016; Figure 1). Consistent with their respective evolutionary history and current sequence similarities, Satellites are in some ways behaviorally and phenotypically intermediate to Independents and Faeders. It follows that divergent regions of the inversion should contain genes or regulatory elements that help explain why certain traits are shared by only two morphs (i.e., Independents and Satellites or Faeders and Satellites). For example, only Satellites and Independents engage in courtship in the lekking competition, but both Satellites and Faeders have a similar androgenic profile and response range, relatively larger testes sizes, and show an adaptive decrease in aggression. Yet, in other aspects, such as body size, pituitary progesterone receptor and gonadal *STAR* gene expression, however, Satellites occupy an intermediate position between Independents and Faeders (Jukema and Piersma, 2006; Küpper et al., 2016; Loveland et al., 2021). Consequently, Satellites provide a unique opportunity to test how recent recombination has affected gene expression variation between morphs. Furthermore, the genetic basis for androgenic differences between inversion morphs has been suggested to derive from expression differences in the *HSD17B2* (hydroxysteroid 17-beta dehydrogenase 2) gene (Küpper et al., 2016) precisely because the enzymatic function of this protein is to convert testosterone to androstenedione (Küpper et al., 2016; Loveland et al., 2021).

In this study, we compare gene expression among ruff male morphs. We sampled ruffs during the breeding season, as this is the time when morph differences in behavior, hormone profiles and appearance are most pronounced. We focused on genes associated with hormone synthesis and/or signaling, located both inside and outside of the inversion, and assayed three tissue types: gonads, adrenal glands and liver. We chose gonads and adrenals because they are major sources for the synthesis of steroid hormones and their precursors, and are involved in the regulation of aggression during breeding and non-breeding contexts (Heimovics et al., 2018). We selected the liver because it is responsible for steroid metabolic processing and can provide information on peripheral mechanisms to offset high circulating testosterone (Bentz et al., 2019). In addition, the liver has proven useful to detect the emergence of gene expression differences in sexual dimorphism during ontogeny (Cox et al., 2017; Cox, 2020).

First, we asked whether genes from the inversion that were previously suggested as candidates for morph-specific traits and fitness (Küpper et al., 2016) differ in expression between inversion morphs and Independent males across tissues, and we tested for allelic imbalance in a subset of these genes in the heterozygous inversion morphs. We hypothesized that the *CENPN* gene, which is interrupted at the proximate breakpoint in inversion carriers, would show reduced expression from the inversion allele relative to the ancestral allele. The *CENPN* gene is the major candidate gene for homozygous lethality of the ruff inversion because it forms part of the constitutive centromere-associated network (CCAN), a protein complex crucial for mitotic centromere assembly (Cheeseman and Desai, 2008; Carroll et al., 2009; Küpper et al., 2016; Yan et al., 2019).

We further predicted that the *HSD17B2* gene, the major inversion-based candidate gene to explain observed morph differences in circulating testosterone and androstenedione, would have higher gonadal expression in inversion morphs because it is the sole enzyme responsible for this enzymatic step. Second, we investigated whether gonadal expression of two key inversion genes, *HSD17B2* and *SDR42E1* (short chain dehydrogenase/reductase family 42E, member 1), in the context of a dozen non-inversion genes involved in steroid synthesis and signaling, could point to specific steps or genes in testosterone synthesis, or steroid hormones at large, that may be atypical in inversion morphs. We selected *SDR42E1* for several reasons. First, inversion morphs share three major deletions surrounding *HSD17B2* and *SDR42E1* genes, which makes a case for gene expression changes due to the elimination or disruption of cis-regulatory elements more likely (Küpper et al., 2016; Lamichhaney et al., 2016). One of these deletions is 5.2 kb in size and located in between *SDR42E1* and *HSD17B2*, genes that are transcribed in opposing directions.

Second, SDR42E1 belongs to the so-called “extended” family of short-chain dehydrogenases/reductases (SDRs) superfamily of enzymes (Persson et al., 2009) and its proposed functions are binding, oxidoreductase activity and 3-beta-hydroxy-delta5-steroid dehydrogenase activity (NCBI AceView database; Thierry-Mieg and Thierry-Mieg, 2006). This last proposed function is ascribed because it contains typical HSD3β domains that could confer enzymatic activity that is partially or fully redundant with other HSD3β enzymes, such as HSD3B2. HSD3B2 is a major enzyme involved in the biosynthesis of all steroid hormones, including metabolizing pregnenolone to progesterone (Albalat et al., 2011), as well as DHEA (dehydroepiandrosterone) to androstenedione. Thus, previously SDR42E1 was speculated to regulate progesterone synthesis (Lamichhaney et al., 2016) and by extension, possibly also androstenedione synthesis. Intriguingly, Faeders have reduced expression of the progesterone receptor gene in the pituitary compared to Independents (Loveland et al., 2021) meaning that differential *SDR42E1* expression could alert toward a possible role for progesterone in explaining morph differences.

Third, with regards to allelic expression, we predicted that due to greater sequence similarity between Independents and Satellites in recently recombined regions, genes in these areas (*ZFPM1, ZDHHC7, ZFN469*) would be similarly expressed in Independents and Satellites, but differently in Faeders. For example, Faeders have 12 and 49 non-synonymous mutations in the *ZFPM1* and *ZNF469* genes, respectively, that are unique to the Faeder haplotype (Küpper et al., 2016). In contrast, for the inversion gene *SPATA2L*, which is located in an area that has high genetic differentiation between Independents and both inversion morphs, we predicted similar expression in Satellites and Faeders versus Independents.

The analysis of inversion genes’ expression offers opportunities to begin to disentangle the mechanistic relationship between function in hormone producing tissues and their phenotypic consequences at the organismal level. Genes that contain androgen response elements, which are short palindromic sequences where androgen receptors bind to regulate transcription, are regulated by androgens including testosterone and its metabolites (i.e., dihydrotestosterone). Given the order of magnitude difference in circulating testosterone levels between Independents and inversion morphs (Küpper et al., 2016; Loveland et al., 2021), we expected that testosterone regulated genes should have overall lower expression in inversion morphs. In addition, because co-expression patterns between genes often indicates they are also functionally associated, we expected that correlations between the expression levels of pairs of genes in Independents, whether positive or negative, might be weakened or absent in inversion morphs.

## Materials and Methods

### Birds and Housing

We sampled 35 adult ruff males (17 Independents, 9 Satellites, 9 Faeders) from a captive breeding flock at Simon Fraser University. The mean age ± standard error (SE) was 5.11 ± 0.4 yrs. This captive population was originally established from eggs collected near Oulu, Finland, in 1985, 1989, and 1990 (Lank et al., 2013). For at least 3 weeks prior to sample collection, males were group-housed in same-sex pens with visual access to females in an outdoor aviary with unrestricted access to food and water and an area to bathe. All housing and procedures (permit #1232B-17) were approved by the Animal Care Committee of Simon Fraser University operating under guidelines from the Canadian Council on Animal Care.

### Tissue Collection and Hormone Analysis

We monitored males to select ones that exhibited their respective morph-specific behaviors according to previously established ethograms (van Rhijn, 1973; Lank et al., 1999) and their behavior was video recorded immediately before killing. No pharmacological manipulations of any kind were performed prior to tissue collection. We collected tissue samples during the breeding seasons of 3 years: 2017 (June 11–17), 2018 (June 5–16), 2019 (June 8–13). We sampled birds in the morning between 8:00–12:15, except for two birds in 2019 that were sampled at 13:00 and 15:00, respectively. Sampling details have been described by Loveland et al. (2021). We dissected samples of the right liver lobe, left and right adrenal glands in their entirety, and left and right gonads. All tissues were rinsed briefly in phosphate-buffered saline (PBS) and dried with a paper tissue before preserving in RNAlater (Ambion) according to manufacturer’s instructions. We weighed gonads to calculate gonadosomatic index (GSI = gonad mass x 100/body mass) before storing one gonad in RNAlater, and the other was frozen in Neg50 medium (Thermo Fisher Scientific) or in aluminum foil on dry ice; the gonad preserved in RNALater was used for RNA extractions. These samples were kept at 4°C for 1–3 days and then stored frozen at −20°C until RNA extraction at the Max Planck Institute for Ornithology in Seewiesen, Germany. We measured testosterone levels from plasma, described by Loveland et al. (2021).

### RNA Extraction and cDNA Synthesis

**FIGURE 2:**
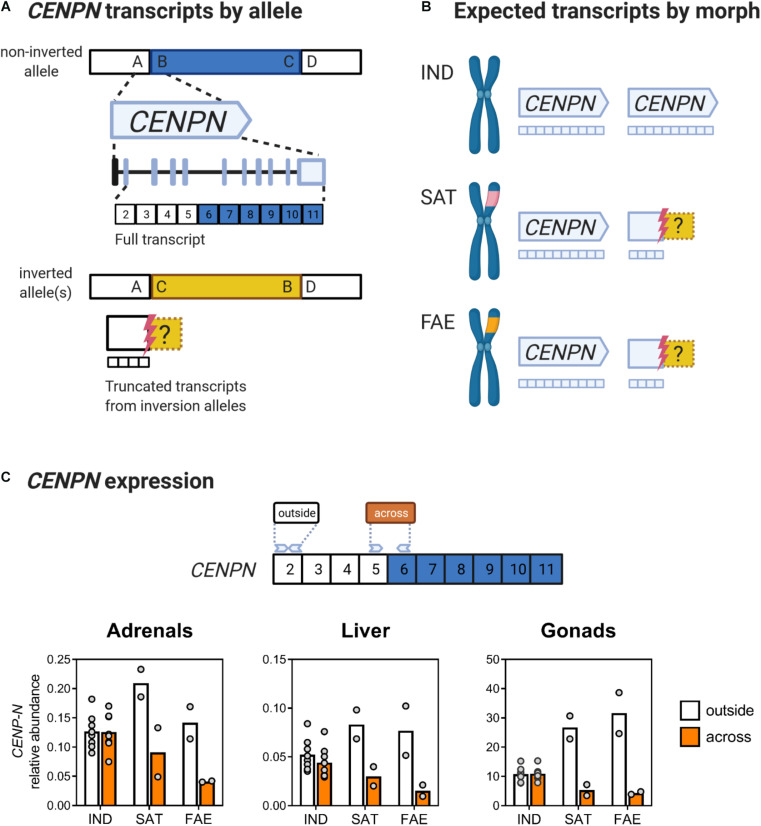
The inversion breakpoint gene *CENPN* has reduced expression in inversion morphs, across tissues. **(A)** Schematic of the order of DNA segments (A-D) on the ancestral allele. The B-C segment (blue) corresponds to the 4.5 Mb inversion region. Segment A flanks the proximal breakpoint, and D flanks the distal breakpoint. The *CENPN* gene spans across the proximal breakpoint and the full transcript contains 11 exons, with the first exon being a non-coding UTR (black). Exons are colored based on their position relative to the corresponding proximal breakpoint: white for outside and blue for inside the corresponding region that becomes inverted. In the inversion allele(s) the order of DNA segments is changed to A, C, B, D with the inversion segment C-B (yellow) reversed from its ancestral orientation. The proximal breakpoint (red bolt) interrupts the *CENPN* gene between exons 5 and 6, thus a truncated four-exon *CENPN* transcript is expected from inversion alleles. Uncertainty about where transcription stops continuing into the inversion is denoted by the dashed yellow box. Exon structure redrawn from NCBI *Calidris pugnax* Genome Viewer. **(B)** Independents produce full *CENPN* transcripts from two ancestral alleles, whereas inversion morphs, which are always heterozygotes for the inversion, are predicted to produce full transcripts from one ancestral allele and only truncated transcripts from their respective inversion allele. **(C)** *CENPN* expression in adrenal glands, liver and gonads for all three morphs plotted as abundance relative to two reference genes (see section “Materials and Methods” for details). In Independent males, expression of exon 2 (white), which is outside of the inverted region, and exons 5 and 6 (orange), which span across the breakpoint, are near identical, whereas in Satellites and Faeders, expression of exons 5 and 6 is much lower than that of exon 2. The amplicon containing exons 5 and 6 spans across the breakpoint and is thus only present on the non-inverted allele in inversion morphs. Sample sizes for adrenals and liver were (8 Independents, 2 Faeders and 2 Satellites) and for gonads were (7 Independents, 2 Faeders, and 2 Satellites). Created with BioRender.com.

**FIGURE 3:**
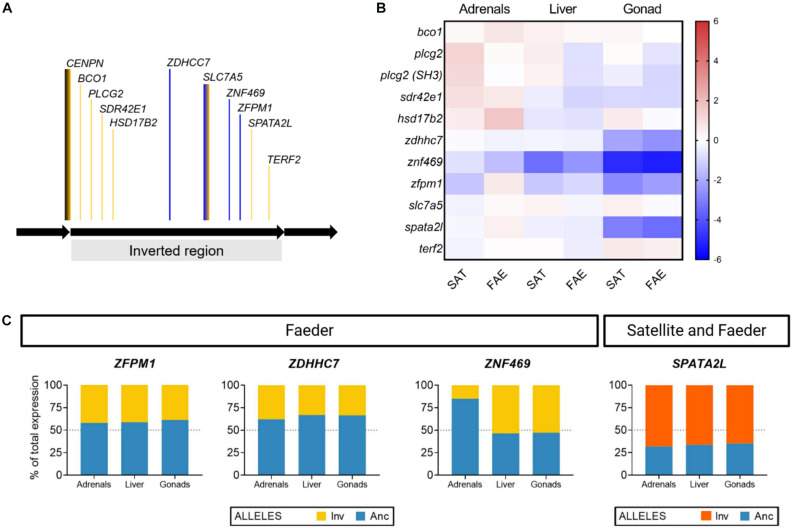
Tissue-specific expression and allelic imbalance of inversion genes. **(A)** Relative location of genes (not drawn to scale) within the inversion that were examined in this study, shown in the orientation of the ancestral non-inverted allele. The line color indicates whether a given gene is in a region with high genetic differentiation between Independents and Satellites (yellow) or high genetic differentiation between Satellites and Faeders (blue); therefore, blue regions correspond to recently recombined regions within the Satellite inversion. *CENPN* spans across the proximal breakpoint (black and yellow line) and *SLC7A5* spans across regions from the most ancient inversion as well as recently recombined areas (blue and yellow line). **(B)** Heatmap visualization of the expression of inversion genes in inversion morphs (Satellites *N* = 2, Faeders *N* = 2), color shading scale indicates increased (red) or reduced expression (blue) relative to Independents (*N* = 8). **(C)** Allelic imbalance in *ZFPM1, ZDHHC7, ZNF469*, and *SPATA2L* genes varies in magnitude, direction and tissue type. *ZFPM1, ZDHHC7, ZNF469* are located in regions that underwent recombination and created the Satellite allele. For each bird, we calculated the contribution of expression from each allele (inversion and ancestral) to the total expression of each gene, and plot the means for each inversion morph (Satellites, *N* = 2; Faeders, *N* = 2) as percent of total expression. In Faeders, both *ZFPM1* and *ZDHHC7* show reduced expression originating from the inversion allele of similar magnitude in all three tissues. In contrast, *ZNF469* had reduced expression of the inversion allele only in the adrenal glands. *SPATA2L* showed increased expression of the inversion allele in all three tissues of both Satellites and Faeders.

We extracted RNA from adrenal gland and liver samples and gonad using the RNeasy Minikit with modifications as described by Loveland et al. (2021). In addition, for adrenal glands, we mixed 200 μl of the lysed homogenate with 400 μl lysis buffer before proceeding with the steps in the standard protocol. This modification to the protocol was performed to prevent possible clogging of the column and to allow preservation of lysed homogenate for future extractions. We measured RNA concentration with a NanoDrop and assessed RNA quality with the Bioanalyzer RNA nanochip (Agilent). Only samples with a 260/280 ratio of ≥ 1.8 and RINs ≥ 5 were used. We excluded four gonad RNA samples from further analysis due to low yield. The average RINs for RNA from gonads, adrenals and liver were 7.4, 8.8, and 7, respectively. For all tissues, we synthesized 1 μg of RNA into cDNA using the iScript cDNA synthesis kit (Bio-Rad) in 20 μl reactions according to manufacturer’s instructions. For comparisons of expression of genes located within the inversion in adrenals, gonads and liver, all cDNA synthesis was performed on the same number of samples (8 Independents, 2 Satellites and 2 Faeders) in 2018. In a second experiment performed in 2019, cDNA was freshly synthesized from gonadal RNA (14 Independents, 8 Satellites, 9 Faeders) to analyze the expression of two inversion genes (*HSD17B2* and *SDR42E1)* along with 12 genes associated with sex hormone synthesis and signaling. We always diluted cDNA 10-fold before use as template in qPCR assays. Sample sizes for tissue RNA extractions for data presented in Figures 2, 3 were 8 Independents 2 Satellites and 2 Faeders; and in Figures 5, 6 were: 17 Independents, 9 Satellites, and 9 Faeders; see notes above for exclusions prior to cDNA synthesis based on RNA yield.

### Primer Design

**FIGURE 4:**
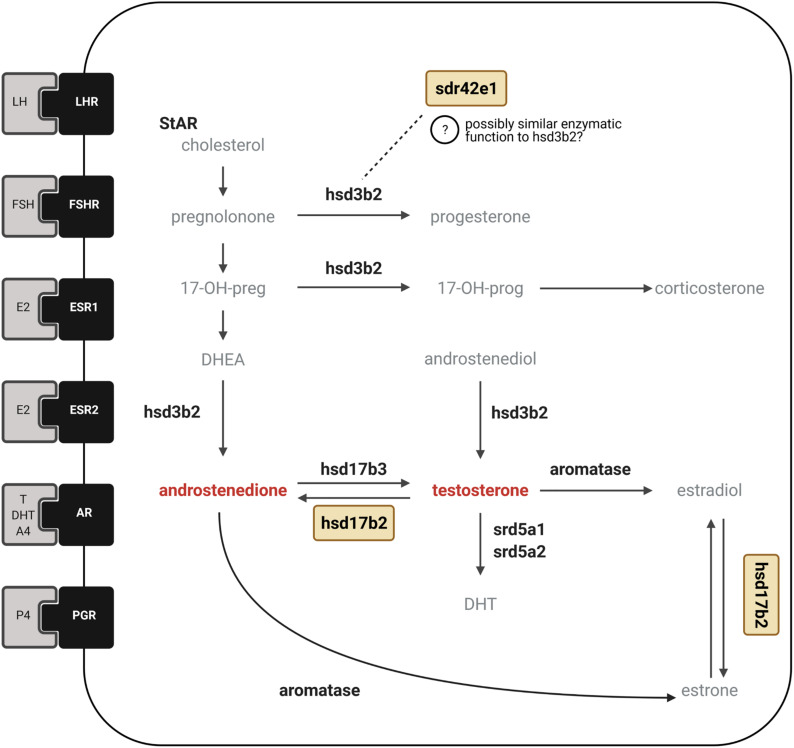
Candidate genes involved in sex hormone synthesis and signaling. Simplified diagram showing the major enzymes involved in pathways for synthesis of steroid hormones with receptors for sex hormones and gonadotropins depicted as membrane-bound. The two inversion genes that encode HSD17B2 and SDR42E1 proteins are shown in yellow boxes. In the ruff, circulating levels of testosterone are drastically reduced in inversion males compared to Independent males. Conversely, inversion morphs have greater levels of androstenedione than Independent males. The *HSD17B2* gene is located in the inversion region and is responsible for the back conversion of testosterone to androstenedione, as well as estradiol to estrone, and has been suggested to be an important candidate that could explain androgenic hormonal differences among ruff morphs (Küpper et al., 2016; Lamichhaney et al., 2016). The *SDR42E1* gene encodes an oxidoreductase enzyme with unknown substrate specificity (indicated with question mark) but has been suggested to have potentially similar enzymatic activity as the HSD3B2 enzyme in progesterone synthesis (Lamichhaney et al., 2016) due to conserved domains characteristic of HSD3β short-chain dehydrogenase enzymes. We measured the expression of 14 genes associated with sex hormone synthesis and signaling encoding six hormone receptors LHR, FSHR, ESR1, ESR2, AR, PGR*;* six steroidogenic enzymes shown in bold, HSD3B2, HSD17B2, HSD17B3, aromatase, SRD5A1, SRD5A2. Created with BioRender.com.

To examine whether male morphs differ in gene expression across tissues, we selected 11 genes with morph-specific SNPs within the inversion to analyze allele-specific expression across three tissue types (gonads, liver and adrenal glands). Details on *CENPN* gene structure are shown in Figure 2 whereas the inversion genes we selected are depicted in Figure 3. We selected an additional 12 non-inversion genes associated with sex hormone synthesis and signaling to analyze expression in gonads (Figure 4). All primers were designed in PrimerBlast using Genbank ruff coding sequences (CDS) as queries; for accession numbers and primer details see Supplementary Table 1 and Loveland et al. (2021).

### Primer Design for Genes Within the Inversion

We designed allele-specific primers such that the target amplicons spanned at least one exon-exon boundary when possible. We manually annotated exon boundaries by a combination of the following: CDS as queries against the chicken genome (v5.0) with the BLAT tool (Kent, 2002) in the UCSC genome browser (Kent et al., 2002); assessment of Augustus v3.1 gene predictions for chicken orthologs and for ruff (NCBI Calidris pugnax Annotation Release 100, software version 6.5). For BLAT results, exon-exon boundaries were called based on their agreement between ruff and chicken. In case of discrepancies between the predicted site of a boundary, we applied the chicken annotation and designed the primer pair to not anneal directly across the boundary, since the exact location was uncertain. For each gene that had morph-specific SNPs, we designed one primer (“common primer”) to anneal to ancestral and inversion haplotypes and a second primer to be allele-specific (Supplementary Table 2). We extracted sequence data from inversion contigs generated from genome assemblies and mapping data reported in Küpper et al. (2016), which were based on high coverage sequencing (>80×) of two individuals of each morph. We confirmed specificity of allele-specific primer pairs by testing them on liver-derived cDNA from one individual of each morph, by assessing melt curves of amplicons and cycle thresholds. Standard curves were performed on serially diluted cDNA in the range of 1:5–1:100. All allele-specific primers showed amplification efficiency for the target allele in the standard curve above 1.9, but no amplification or low efficiency (<1.3) with unambiguously late amplification in the exponential phase of the common allele when the target allele was absent; an example is shown in Supplementary Figure 1. The details for the design for *CENPN* primers that span across the inversion breakpoint are given in Figure 2A. For five inversion genes (*CENPN, BCO1, SLC7A5, PLCG2, TERF2)* that did not involve allele-specific measurements across tissues we assumed an amplification efficiency of two for all morphs; for all other genes we used gene efficiencies from standard curves.

### qPCR Conditions

We performed qPCR experiments with SsoAdvanced Universal SYBR Green Super mix (Bio-Rad) as described in Loveland et al. (2021) with the modification that for inversion genes and allele-specific assays, the qPCR was run for 40 cycles. A report of efficiencies for all primer pairs corresponding to non-inversion genes not published in Loveland et al. (2021) is given in Supplementary Table 3. For all experiments, we ran each sample in duplicate and used the following conditions: 10 μl reaction with 1X SYBR mix, 400 nM of each primer and 7 ng of cDNA template. To control for interplate effects, each plate contained samples of Independent, Satellite and Faeder males and both reference and target genes were assayed on the same plate. To test for any possible genomic carryover from RNA extraction to cDNA synthesis, we ran the qPCR with cDNA synthesis negative controls (i.e., no reverse transcriptase) for a subset of samples (24 of 36 samples in 2018); none showed any amplification.

### Relative Abundance

We calculated relative abundance as the expression of target genes relative to two reference genes as described in Loveland et al. (2021). For the qPCR experiments involving only inversion genes on cDNA from gonads, liver and adrenals, we used *GAPDH* and *RPL30* as reference genes, whereas for qPCR experiments involving *HSD17B2* and *SDR42E1* with the 12 non-inversion gene set on cDNA from gonads, we used the reference genes *HPRT1* and *RPL32.*

### Statistics

The *CENPN* gene is interrupted by the inversion and has been proposed as the main reason why the inversion is homozygous lethal (Küpper et al., 2016). To test whether morphs differed in the relative abundance of *CENPN* transcripts from “outside” (present in all alleles) vs. “across” the proximal inversion breakpoint (present in ancestral allele only), we divided expression of the “across” fragment by the expression of the “outside” fragment. As both amplicons are present in the full ancestral transcript we expected this ratio to equal “1” in Independents and conversely, “0.5” in inversion morphs due to the “across” amplicon being absent in inversion alleles (Figure 2B). We tested the mean ratio of each group (i.e., Independent and inversion morphs) with a one-sample *t*-test against a hypothesized mean of one, which assumes that only full transcripts from the ancestral allele are being expressed. In addition, we used the “across” amplicon as an estimate of the abundance of non-truncated *CENPN* transcripts, and compared Independent vs. inversion morph means with a Mann-Whitney U-test, given limited sample sizes. To test allelic imbalance for genes *ZFPM1, ZDHHC7, ZNF469, SPATA2L* we asked whether the contribution of expression from the inversion allele to the total levels (i.e., ancestral allele amplicon + inversion-specific amplicon) differed from 50% with a one-sample t-test. To correct for multiple testing (4 genes, 3 tissues) we applied a Bonferroni-Dunn correction.

To generate the heatmap visualization of the expression of genes from the inversion we calculated for each gene, the mean expression in Independents divided by the expression in an individual inversion morph bird. The means for Satellites and Faeders were then plotted separately on a logarithmic (log2) scale. As, except for *CENPN*, there were no statistically clear expression differences across tissues, we used a heatmap to visualize the results on how expression differed across tissues and morphs.

**FIGURE 5:**
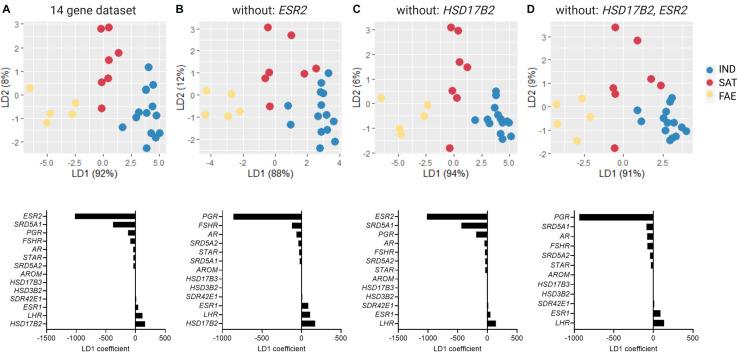
Linear discriminant analysis (LDA) of the testicular expression of 14 genes associated with sex steroid metabolism and signaling, including inversion genes *HSD17B2* and *SDR42E1*, for Independents (*N* = 13), Satellites (*N* = 7), and Faeders (*N* = 5). Each panel shows an LDA plot with its corresponding LD1 coefficients. **(A)** LD1 explained 92% of the variance in the full 14 gene dataset. Loading was most heavily influenced by the expression differences in *ESR2* and *SRD5A1* (negative loadings) and in *HSD17B2* and *LHR* genes (positive loadings). **(B)** The separation between Independents and Satellites was lessened, but not completely lost, after the removal of the gene with the largest negative loading (*ESR2*), whereas alternatively, **(C)** removal of the gene with the largest positive loading (*HSD17B2*) still provides clear separation between these two morphs. **(D)** Removal of both *ESR2* and *HSD17B2* results in overlapping of Independents and Satellites along LD1, as in **(B)**.

To collectively analyze the expression of genes associated with sex hormone synthesis and signaling, we performed linear discriminant analysis (LDA) in the R package (MASS) with morph as a preset class, and included the full 14 gene dataset (Figure 5A and Supplementary Figures 3A, 4A). With this analysis we identified axes loadings that explain most of the variation between morphs, such that the separation between morphs is maximized while the variation within morphs is kept minimal. First, we analyzed two 13 gene datasets, each with one of the two top genes with heaviest loadings removed (*ESR2* or *HSD17B2*) (Figures 5B,C) and one 12 gene dataset with both *ESR2* and *HSD17B2* removed (Figure 5D). We then proceeded to remove in step-wise fashion the top two genes with highest positive and negative loadings on LD1 and ended with three genes as the last dataset (Supplementary Figures 3B–L). This approach showed how robust morph clustering was to the removal of strongly influential genes. In the second approach, we instead removed in a step-wise fashion genes that had the lightest positive and negative loadings to arrive at the minimal dataset that would preserve morph clustering (Supplementary Figures 4B–H).

To test for morph-specific correlations of gene expression, we calculated correlation matrices for each morph on the 14 gene dataset (i.e., sex hormone synthesis and signaling genes including two inversion genes *HSD17B2* and *SDR42E1*), plus circulating testosterone and GSI values. We expected this analysis would identify genes with expression associated with testosterone, as activated androgen receptor translocated into the nucleus regulates the expression of genes that contain androgen response elements in their respective regulatory regions (Tan et al., 2015). In addition, we tested for correlations of gene expression with GSI, to explore potential links with spermatogenesis. We predicted that correlations between gene expression and testosterone in Independents would be absent in inversion morphs as these have lower testosterone levels. Similarly, we predicted differences in co-expression between our candidate genes between Independents and inversion morphs. This analysis rendered a total of 360 correlations. The corresponding *p*-values were adjusted with a False Discovery Rate (FDR) of 10% with the Benjamini and Hochberg method (Benjamini and Hochberg, 1995). We used GraphPad Prism (version 8) and R (version 3.6.2) for statistical analyses.

## Results

### *CENPN* Expression

For Independent males the relative abundance of the “across” amplicon of the *CENPN* gene was not different from the relative abundance of the “outside” amplicon in gonads (*t*_6_ = 0.5, *p* = 0.63) and adrenal glands (*t*_7_ = 0.16, *p* = 0.88), but showed a statistically clear difference in the liver (*t*_7_ = 3.06, *p* = 0.02) (Figure 2C). In contrast, in inversion morphs, the relative abundance of the “across” amplicon was always lower in all tissues, with gonads (*t*_3_ = 34.92, *p* < 0.001), liver (*t*_3_ = 14.47, *p* < 0.001), and adrenals (*t*_3_ = 8.63, *p* = 0.003) often not even reaching 50% of the “outside” amplicon expression (Figure 2C). Using the “across” amplicon as an estimate of levels of non-truncated *CENPN* transcripts, inversion morphs had reduced expression compared to Independents, that was statistically clear in gonads (Mann-Whitney *U* = 0, *p* = 0.006) and liver (Mann-Whitney *U* = 4, *p* = 0.048), but not quite in adrenal glands (Mann-Whitney *U* = 5, *p* = 0.07).

### Expression of Inversion Genes and Allelic Imbalance Across Tissues

Overall, there were no clear morph differences in the expression of inversion genes other than *CENPN*, but we note that because of small sample sizes for these particular assays (7–8 Independents, 2 Satellites, 2 Faeders) the lack of clear differences should be viewed with caution. Nonetheless, across the three tissues, the gonads showed the most pronounced decreases in expression of inversion morphs compared to Independents (Figure 3B). We found evidence of allelic imbalance (AI) in expression from the inversion allele(s) for *ZFPM1, ZDHHC7, ZNF469*, and *SPATA2L* genes. The degree of AI appeared to vary in magnitude, direction and by tissue type (Figure 3C and Supplementary Table 4) although sample size limitations especially for Faeders (*N* = 2) need to be taken into account. Nonetheless, after correcting for multiple testing, increased expression from the inversion allele for *SPATA2L* in adrenals (*t*_3_ = 9.715, Bonferonni-Dunn adjusted *p* = 0.028) and liver (*t*_3_ = 8.51, Bonferonni-Dunn adjusted *p* = 0.041) were statistically clear for inversion morphs (2 Satellites and 2 Faeders).

### Testicular Expression of Inversion Genes *HSD17B2* and *SDR42E1* in the Context of Sex Hormone Synthesis and Signaling Pathways

Consistent with previous reports (Küpper et al., 2016), morphs differed in GSIs [ANOVA *F*_(2, 32)_ = 8.44, *p* = 0.001] with Faeders having clearly higher GSI compared to Satellites (Tukey’s *p* = 0.019) and to Independents (Tukey’s *p* < 0.001), and no clear difference between Independents and Satellites (Tukey’s *p* = 0.63) (Supplementary Figure 2). All males had enlarged gonads typical of expected sizes during the breeding season, but there were no statistically clear differences among morphs (Supplementary Figure 2). Because the analysis did not allow missing values and we were forced to exclude individuals that did not have the full 14 gene data set, the sample sizes were reduced to 13 Independents, seven Satellites and five Faeders. The combined gene expression of 14 genes associated with sex steroid metabolism and signaling showed clear separation of the three morphs (Figure 5A). LD1 explained 92% of the variance and was most heavily loaded positively by the *HSD17B2* and *LHR* genes and negatively by the *ESR2* and *SRD5A1* genes. Only the clustering of Faeders was robust to removal of individual genes (Supplementary Figure 3). Removal of genes with the heaviest loadings (*ESR2* and *HSD17B2)* affected this separation when *ESR2* was the only one removed, or when both were removed at the same time (Figures 5B,D). However, removal of the inversion gene *HSD17B2* did not affect the separation among the three morphs (Figure 5C). Subsequent sequential removal of two genes with the heaviest loadings at a time did not greatly disturb the Faeder cluster (Supplementary Figure 3). Only after the removal of the top 11 genes with the heaviest loads, morph separation was not possible. Full results of the sequential removal of genes with heaviest loadings are provided in Supplementary Figure 3. In the second sequential analysis, we removed pairs of genes with the lightest loadings to discover the minimal core number of genes in the dataset that retains morph separation. This showed that at least 10 genes were necessary to retain all three clusters (Supplementary Figure 4).

**FIGURE 6:**
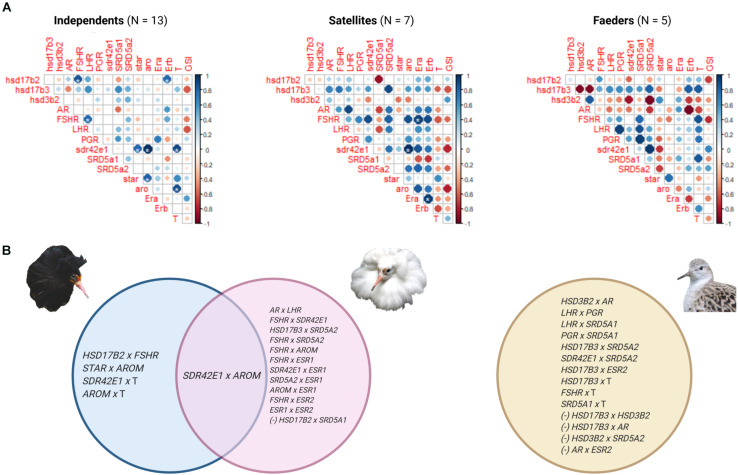
Gene expression correlations in testes by morph. **(A)** Correlation matrices of the expression of 14 genes, circulating testosterone (T) levels and gonadosomatic index (GSI) for each morph. The color of the circle illustrates the sign of the correlation (positive, blue; negative, red) and size of the circle represents the strength of the correlation. An asterisk indicates significant correlations that had a false discovery rate adjusted threshold, *p* < 0.003: Independents and Satellites had 8 and 3, respectively. Genes *ESR1* and *ESR2* are abbreviated as Era and Erb, respectively. **(B)** Venn diagram depicting gene pairs that had a correlation coefficient ≥0.8 or ≤–0.8 in Independents (blue), Satellites (pink), and Faeders (yellow). Expression of the inversion gene *SDR42E1* and *AROM* gene was strongly positively correlated in both Independents and Satellites, but not in Faeders. *SDR42E1* expression was positively correlated with testosterone levels in Independents only. Negative correlations (–) between pairs of genes were only observed in inversion morphs. Created with BioRender.com.

Pairwise correlations in the expression of genes rendered significant correlations for 18, 15 and 9 pairs in Independents, Satellites and Faeders, respectively. After the FDR-adjustment, 8 of these correlations in Independents and 3 in Satellites remained significant; all were positive correlations (asterisks in Figure 6A matrices). Of these, only one pair comprising the inversion gene *SDR42E1* and *AROM* was shared between Independents and Satellites (Figure 6B). Interestingly, these two genes were also the ones with the highest correlation coefficients within these two morphs (Independents *r* = 0.99, FDR-adjusted *p* = 5.36 × 10^–9^; Satellites *r* = 0.99, FDR-adjusted *p* = 3.3 × 10^–6^). Because biologically relevant correlations may not reach statistical significance with smaller sample sizes compared to larger ones, and in our study Independents had the largest sample size, we list all pairs of genes that had correlation coefficients ≥0.8 (≤–0.8), regardless of *p*-values (Figure 6B).

## Discussion

We used a quantitative PCR approach on candidate genes located inside and outside a prominent autosomal inversion to assess how the stable inversion polymorphism in male ruffs affects gene expression across three different tissues. Two of the tissues, adrenal glands and gonads, are important sources for hormones, and the liver is responsible for further processing steroid metabolites. Our candidate genes were either involved with steroid hormone synthesis and signaling, or major transcription factors chosen because they regulate the expression of many genes, both inside and outside of the inversion. We present evidence for decreased expression of the *CENPN* gene in inversion morphs, as well as allelic imbalance and morph-specific variation in the collective expression of genes associated with hormone synthesis and signaling.

### Reduced Expression of the Breakpoint Gene *CENPN*

We first examined the expression of inversion genes across tissues in all three morphs, including the *CENPN* gene, a major candidate for the homozygous lethality of the inversion because it is interrupted by the proximal breakpoint in both inversion morphs (Küpper et al., 2016). Consistent with dosage dependent expression, we found that in Independents, transcript regions from outside and across the breakpoint showed similar levels of expression, whereas in both Satellites and Faeders, the expression of the transcript regions interrupted by the breakpoint was reduced by a factor of two or more. A qualitative assessment of *CENPN* expression across tissues showed that its expression was greatest in gonads for all morphs, compared to adrenals and liver, which is likely due to the proliferative nature of gonadal tissue for spermatogenesis during the breeding season. Among all candidate genes investigated, *CENPN* had the strongest pattern of reduced expression in inversion morphs relative to Independents. The CENPN protein plays an essential role in the kinetochore, as it is responsible for making contacts with the nucleosome bound CENPA protein, a first step in linking centromeric chromatin to microtubules of the mitotic spindle (Carroll et al., 2009; Yan et al., 2019). These results are consistent with the hypothesis that the inversion is homozygous lethal because reduced expression from two inversion alleles would be insufficient to sustain cell division. Furthermore, our findings align with previous reports where breakpoint genes in supergene-mediated behavioral polymorphisms are disrupted in a similar manner across inversion genotypes, sexes and tissues (e.g., see LOC105193832 in Yan et al., 2020). Thus, any negative effects of inversions on the expression of genes that span across or near breakpoints represent the very first challenges that must be offset in order for inversions to have the opportunity to persist within populations. In the case of the ruff, it is clear that the ability to achieve and maintain a minimal level of *CENPN* expression from the ancestral allele is a critical element in the evolutionary trajectory of inversion morphs.

### Allelic Imbalance Across Tissues in Inversion Morphs

We found evidence of allelic imbalance in all four inversion genes for which we were able to design allele specific primers that passed all quality control criteria. The magnitude and direction of the imbalance varied by tissue type. Allelic imbalance was consistent across individuals of the same morph, however, we note that sample size was limited, as we only examined four inversion morph individuals (two Satellites and two Faeders). Three genes (*ZFPM1, ZNF469, ZDHHC7*) were in recently recombined areas and therefore SNPs were exclusive to the Faeder haplotype. We therefore predicted that expression levels would be different in Faeders compared to the other two morphs. Contrary to our expectation, there were no differences in total expression between Satellites and Faeders. However, the two Faeders had reduced expression from the inversion allele for major zinc-finger transcription factors (*ZFPM1, ZNF469*) across all tissues. The third gene *ZDHHC7* (zinc finger DHHC-type palmitoyltransferase 7) is essential to the palmitoylation of sex steroid receptors (estrogen receptors, androgen receptor and progesterone receptors) (Marino and Ascenzi, 2006; Pedram et al., 2012) and showed dramatically reduced expression from the inversion allele in the adrenal glands only. ZDHHC7 is required for the ability of the estrogen receptor to exert rapid non-genomic (membrane-bound) effects in the brains of mice (Acconcia et al., 2004; Hohoff et al., 2019). Given that estrogen plays a central role in aggressive and courtship behavior in many species of birds (Soma, 2006), the expression differences in *ZDHHC7* could be biologically relevant. We also observed reduced (albeit not statistically clear) total expression of the *ZDHHC7* gene in gonads among inversion males (Figure 3B) and a reduced expression of Faeder allele compared to the ancestral allele (Figure 3C). We speculate that these differences could affect brain development and adult sexual behavior through a deficit in one or both estrogen receptors, although this requires further studies.

There are ∼39 genes in the recently recombined areas that have high sequence similarity between Satellites and Independents (Küpper et al., 2016; Lamichhaney et al., 2016). Three of these (*ZFPM1, ZNF469, ZDHHC7*) we investigated here and found that gene expression differences seemed unrelated to sequence similarity as Faeder is the divergent morph according to sequence similarity but expression was more similar in Faeders and Satellites with Independents showing different expression for these genes. Follow up studies with larger sample sizes and involving all genes in the areas of recent recombination will shed a light whether this is a general pattern. Nonetheless, our results provide an interesting challenge to the view that gene expression patterns can be predicted based on sequence similarity or level of divergence alone. For the fourth gene (*SPATA2L*), located in an area where Satellites and Faeders share sequence similarity and both share many SNPs with respect to Independents, inversion morphs showed qualitatively reduced gonadal total expression compared to Independents (Figure 3B), but increased expression from their respective inversion alleles across all tissues assayed (Figure 3C). The *SPATA2L* gene is a paralog of *SPATA2*, which is involved in spermatogenesis (Graziotto et al., 1999; Onisto et al., 2000). *SPATA2L* has been reported to have enriched expression in the brains of zebrafish (*Danio rerio)* (Moro et al., 2002; Maran et al., 2009), which makes it an interesting candidate for further analysis in the ruff, as the morphs show pronounced differences in testes volume index (Küpper et al., 2016) and relative testes size (Supplementary Figure 2). Our allelic imbalance results add to previous research that has shown allelic imbalance for another gene within the inversion, *MC1R*, where Satellites have reduced expression from the inversion allele in dark colored feathers, compared to Independents (Schwochow-Thalmann, 2018). We show that across several tissues, expression from the inversion is often reduced, but in some cases may be increased relative to the ancestral allele, as illustrated by *SPATA2L.*

### Morphs Can Be Discriminated Based on Expression Differences of Multiple Genes

We analyzed the expression of two key inversion genes, *HSD17B2* and *SDR42E1*, in the context of a dozen non-inversion genes involved in steroid synthesis pathways and signaling in testes. The goal was to examine whether patterns of co-expression differed between Independents and inversion morphs, and then between Satellites and Faeders as they show differentiated alleles (Küpper et al., 2016). We selected these two particular inversion genes because of their potential to play direct roles in the hormonal differences among morphs (Küpper et al., 2016; Lamichhaney et al., 2016). Morphs show profound differences in androstenedione and testosterone levels during the breeding season, therefore we expected to observe differences in gene expression for *HSD17B2* because this gene’s product catalyzes testosterone into androstenedione. In addition, inversion morphs share three major deletions surrounding *HSD17B2* and *SDR42E1*, with one such deletion located in between the two genes and as a consequence, has the potential to alter their expression (Küpper et al., 2016; Lamichhaney et al., 2016). Despite our expectation, neither of the two key genes showed clear expression differences between morphs. Yet, we found that the expression of *SDR42E1* and aromatase genes were positively correlated in both Independents and Satellites, but not in Faeders. This result points to a putatively novel relationship between the inversion and estrogen synthesis, which is an exciting avenue for future research given the well-documented importance of estrogen in brain organization and courtship behavior in other birds (Steimer and Hutchison, 1980; Fusani et al., 2001; Gahr, 2001; Court et al., 2020). However, because both Satellites and Faeders share the deletion that is upstream from *SDR42E1*, identifying which sequence elements (i.e., mutations unique to the Satellite allele or shared between Independents and Satellites) can account for the Satellite and Faeder differences concerning *SDR42E1* and aromatase co-expression will require further study.

Although we did not find expression differences for inversion genes besides *CENPN*, these results echo a previous study on gene expression from feathers of 5 Independent and 6 Satellite males, where none of approximately 6,000 genes assayed were differentially expressed between morphs (Ekblom et al., 2012). In our analysis of non-inversion genes, we also only found differential expression among morphs for *STAR* in the testes (reported previously in Loveland et al., 2021), but most interestingly we found clear non-overlapping morph-specific gene expression profiles when all 14 genes (2 inversion, 12 non-inversion genes) were analyzed together. Consistent with their genotype and phenotype, Satellites clustered in between Faeders and Independents. Remarkably, neither removal of *HSD17B2* nor *ESR2*, the genes with heaviest positive and negative loadings, respectively, did fully abolish clustering of the three morph groups. *ESR2* may be involved in encoding the behavioral differences between males, as a recent study showed that during embryological development the expression of *ESR2*, and not *ESR1*, mediates the organizational de-masculinization of the brain with permanent effects into adulthood in the Japanese quail (*Coturnix japonica*) (Court et al., 2020). When we sequentially removed genes according to their descending loadings, the separation of Faeders from Independents and Satellites persisted until we reached four genes: *STAR, HSD17B3, HSD3B2, AROM* (Supplementary Figures 3E–H). Subsequent removal of the *STAR* gene, which we previously reported as differentially expressed across morphs (Loveland et al., 2021), led to a collapse of the distinct Faeder cluster (Supplementary Figure 3I). These results show that the inversion gene *HSD17B2* may be important in the greater context of the gene network for steroid synthesis and signaling, as it ranked among the heaviest loadings, but on its own does not show clear morph differences. Notably, even when both inversion genes *HSD17B2* and *SDR42E1* were removed from the dataset, Faeders continued to cluster separately from Independents and Satellites, indicating perhaps more pronounced trans-acting effects on genes outside of the inversion by the ancient Faeder inversion haplotype than the derived Satellite haplotype. This difference between Faeder and Satellite inversions is more likely to be due to sequence divergence that is independent of hormone levels, given the similarity in androgenic profiles between Satellites and Faeders (Loveland et al., 2021). In contrast, the stepwise removal of genes with lightest loadings led to a core of 10 genes where morphs still clustered, whereas with fewer genes the separation became gradually less clear and completely collapsed in the 2 gene dataset (Supplementary Figure 4).

The lack of observable morph differences in the expression of *HSD17B2* does not necessarily rule out a direct role for this gene, as we only measured gene expression and did not directly measure enzymatic activity of this protein. This gene has also accumulated several missense mutations that could affect its function (Küpper et al., 2016; Lamichhaney et al., 2016). One such mutation (A235S) is shared by Satellites and Faeders and is located immediately adjacent to the tyrosine residue (Y236) that forms part of the highly conserved (“N-S-Y-K”) catalytic tetrad of these types of enzymes (Persson et al., 2009, 2003) (numbering based on accession number XP_014797711). Therefore, it is possible that the HSD17B2 enzyme, given evidence of this missense mutation at a critical location, has modified its catalytic rate in inversion morphs compared to Independent males.

We found that the inversion gene *SDR42E1* was strongly positively correlated with aromatase in Independents and Satellites only. Two putative functions for SDR42E1 are oxidoreductase activity and 3-beta-hydroxy-delta5-steroid dehydrogenase activity, which would give it the capacity to affect several intermediate molecules in steroid synthesis, similar to the role of HSD3B2 (see Figure 4). SDR42E1 might be involved in converting DHEA to androstenedione, as well as pregnenolone to progesterone, which would be relevant to the hormonal profiles of inversion morphs. Furthermore, in seasonal breeders, the sequential conversion in the brain of adrenally sourced DHEA into androstenedione by HD3B2, followed be the conversion of androstenedione to testosterone and then to estrogen by aromatase, has been proposed as one possible mechanism to regulate aggression during the non-breeding season in other bird species, when testosterone levels are very low (Heimovics et al., 2018). Given the low levels of testosterone in inversion morphs, it will be of interest to know whether such a mechanism is at play, either mediated by HSD3B2 and/or SDR42E1, and whether it influences the nearly discrete differences in aggression and courtship behavior between morphs. Unfortunately, direct evidence for specific functional roles of SDR42E1 are so far lacking but it certainly remains an appealing candidate for more detailed studies in ruffs. Our report that it has a relationship of co-expression with aromatase, and the fact that *ESR2* ranked in the top three genes in the LDA analysis that was key to separating Satellites from Independents, suggests that estrogen should be given a focus in future studies, given its behavioral effects require the aromatization of androgens. In other bird species, the aromatization of testosterone into estrogen is essential to organizational and activational effects on the brain for adult courtship behaviors (Steimer and Hutchison, 1980; Belle et al., 2005; Soma, 2006; Court et al., 2020), so low testosterone levels in inversion morphs could also have a major consequence on estrogen levels.

Interestingly, *PGR* expression in the testes repeatedly ranked among the top genes with negative loadings. In a recent study, we reported that Faeder males had reduced pituitary expression of *PGR* compared to Independent males (Loveland et al., 2021). Progesterone has been implicated in the regulation of aggression in females in black coucals *Centropus grillii*, a species with reversed sex roles (Goymann et al., 2008), and in courtship behavior in the ring dove (*Streptopelia risoria*), specifically in the context of the effects of aromatase inhibitors (Belle et al., 2005). Thus, it seems that roles for estradiol and progesterone are emerging as potentially relevant for differences among male morphs and it will be interesting to see whether these finding extend into more comprehensive analyses of transcriptomes in neural tissue.

## Conclusion

A chromosomal inversion has contributed to the origin of two male ARTs and their persistence over considerable evolutionary time. Among ruff males, extreme sexual selection has induced a strong mating skew where a few dominant males sire most of the offspring. The inversion enabled two new types of males to exploit a previously unoccupied social niche. In this way, the inversion profoundly altered behavioral phenotypes and the dynamics of the mating competition. Today’s Satellites lack aggression whereas today’s Faeders lack both aggression and courtship. Examining patterns of gene expression provides information on the molecular underpinnings of these seemingly adaptive losses of functions in inversion morphs. The ruff inversion captured several genes involved in steroid metabolism. These provide strong candidate genes for the discrete differences in the reproductive biology between morphs, as steroids are major modifiers of gene expression as their genes are highly pleiotropic. We investigated how the expression of candidate genes associated with viability and, differences in physiology and behavior located both, inside and outside of the inversion varies across tissues in male ruffs. As expected, we found that the breakpoint gene *CENPN* has reduced expression in inversion morphs and given its essential role in cell division, still stands as the most likely reason why the inversion is homozygous lethal. We found widespread evidence across tissues for allelic imbalance in inversion genes that are major transcription factors (*ZFPM1, ZNF469)*, required for the rapid non-genomic effects of estrogen receptors (*ZDHHC7)*, and relevant to spermatogenesis *(SPATA2L)*. Besides *CENPN*, no other inversion gene showed differential expression across morphs. However, analyses of the collective expression of 14 genes (combining inversion and non-inversion genes) produced clear non-overlapping clusters of three morphs. When key inversion genes were analyzed collectively with non-inversion genes related to steroid metabolism pathways and signaling, we discovered that the *SDR42E1* and *AROM* genes were positively correlated in Independents and Satellites, but not in Faeders. These results suggest that estradiol synthesis may also be affected by the inversion and could have important implications for understanding the evolution of cooperative courtship in this species. Our findings for genes in recently recombined regions showed that sequence similarity did not predict gene expression patterns, although future work should clarify whether this is true for other genes in these areas. This study provides the basis for further more detailed investigations using gene network analysis based on full transcriptomes.

## Data Availability Statement

The raw data are stored in Edmond, the Open Research Data Repository of the Max Planck Society (https://dx.doi.org/10.17617/3.5z).

## Ethics Statement

The animal study was reviewed and approved by the Animal Care Committee of Simon Fraser University.

## Author Contributions

JLL and CK conceived the gene expression elements of the project. DBL established and maintained the captive flock. JLL collected and processed samples, performed qPCR, and analyzed the data. JLL wrote the initial draft with input from CK. All authors edited and approved the final manuscript and designed sample collection conditions.

## Conflict of Interest

The authors declare that the research was conducted in the absence of any commercial or financial relationships that could be construed as a potential conflict of interest.
