# Supplementary material for: Gene Expression Modification by an Autosomal Inversion Associated With Three Male Mating Morphs
Source: Front Genet. 2021 Jun 4;12:641620. doi: 10.3389/fgene.2021.641620 (PMC8213371; doi:10.3389/fgene.2021.641620)

Supplementary Figures

Figure S1. Representative example of standard curve results used to assess allele-specificity in ancestral and inversion-specific primers

Screenshots for standard curve results for the *ZDHCC7* gene showing ‘ancestral’ primers that detect expression from the common ancestral allele and those that detect expression from the Faeder inversion. The ‘ancestral’ primers perform with an efficiency above 1.9 with cDNA from an Independent male, as well as with cDNA from a Faeder male. Similarly, Faeder-specific primers amplify with an efficiency above 1.9 when used with Faeder cDNA. The crossing point for these amplicons is in the range of cycles 26-30. In contrast, when cDNA from an Independent male is tested with Faeder-specific primers amplification is inconsistent between duplicate wells, which results in a non-linear plot and an extremely low efficiency (1.3), which is well below our criteria for acceptable efficiency (≥1.9). This indicates that Faeder-specific primers do not amplify cDNA from the ancestral allele, even when cDNA from the Faeder inversion allele is absent. These results paired with melt curve checks were used to deem inversion-specific primers as acceptable or not, therefore only four genes (out of 11 initially tested) passed all criteria.

ZDHHC7

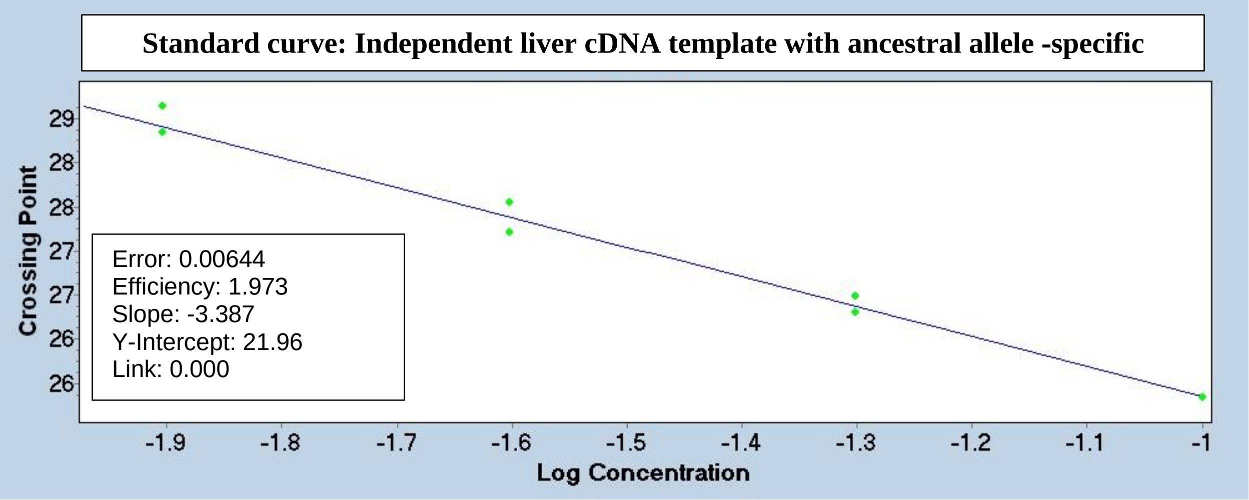

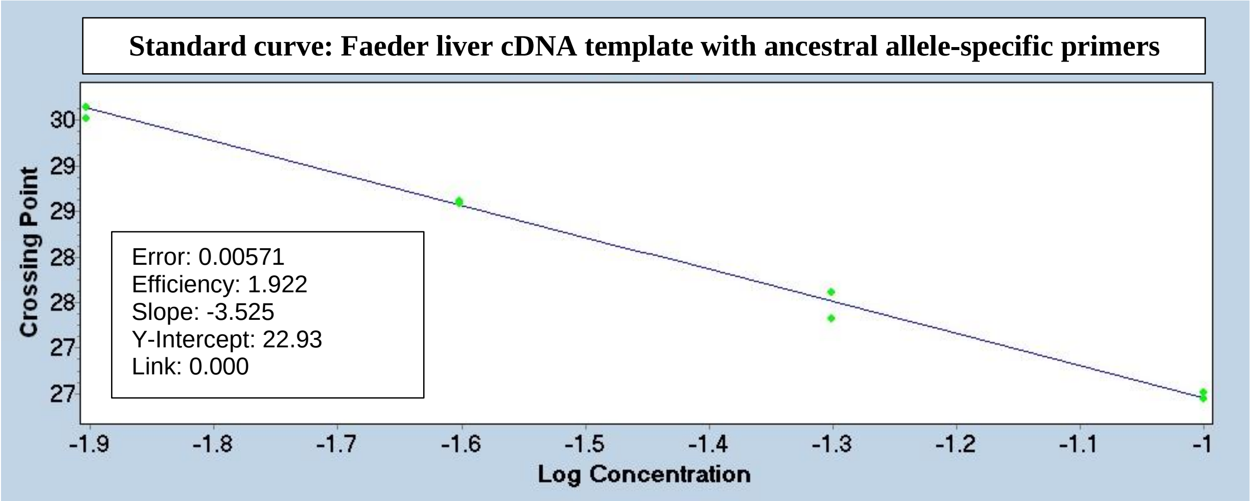
Standard curve: Faeder liver cDNA template with ancestral allele-specific primers
Crossing Point
30
29
29
28
28
27
27
Error: 0.00571
Efficiency: 1.922
Slope: -3.525
Y-Intercept: 22.93
Link: 0.000
-1.9
-1.8
-1.7
-1.6
-1.5
-1.4
-1.3
-1.2
-1.1
-1
Log Concentration

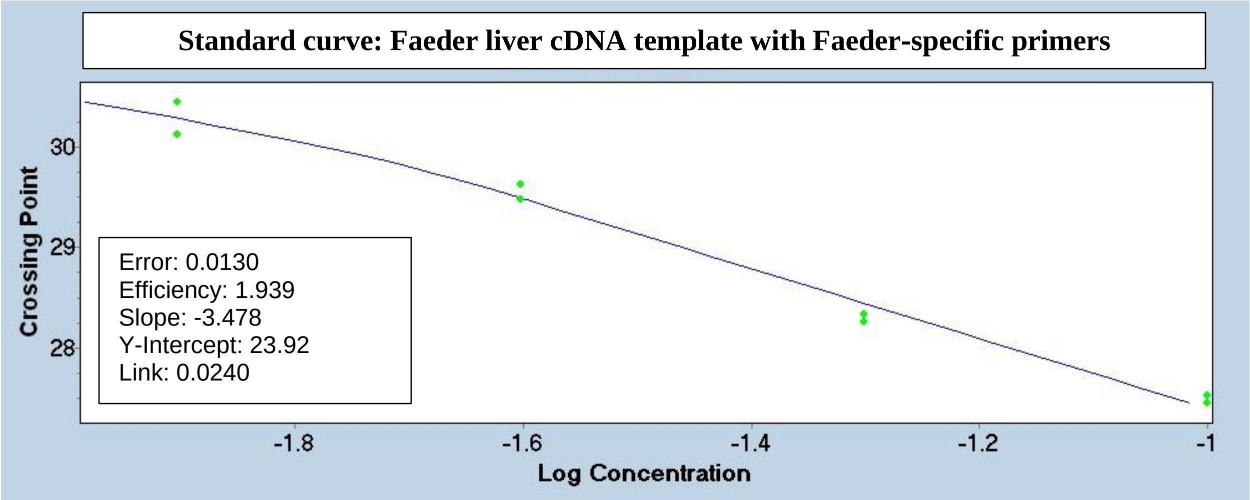
Standard curve: Faeder liver cDNA template with Faeder-specific primers
Crossing Point
30
29
28
Error: 0.0130
Efficiency: 1.939
Slope: -3.478
Y-Intercept: 23.92
Link: 0.0240
-1.8
-1.6
-1.4
-1.2
-1
Log Concentration

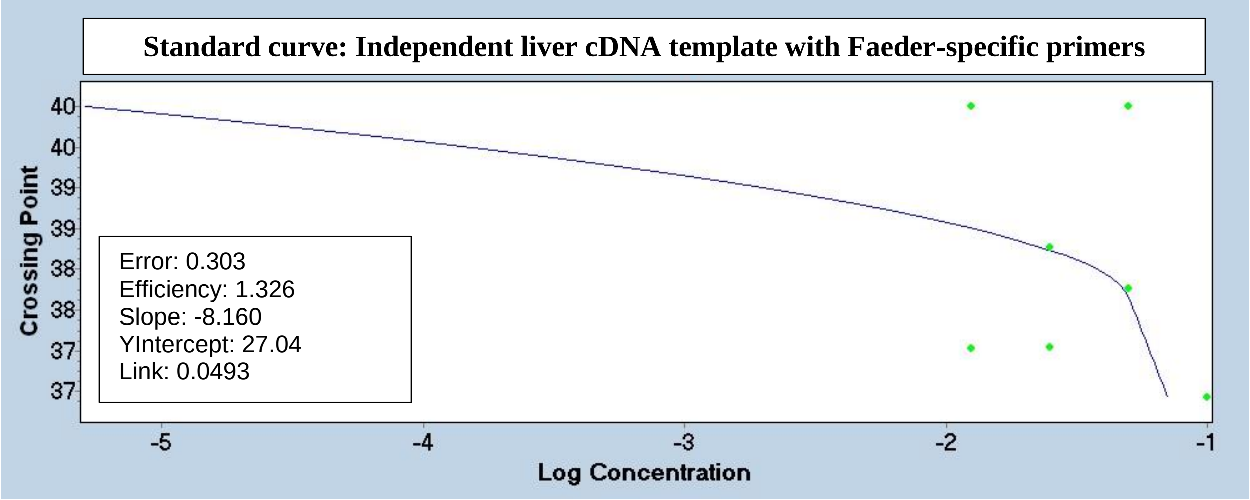
Standard curve: Independent liver cDNA template with Faeder-specific primers
Crossing Point
40
40
39
39
38
38
37
37
Error: 0.303
Efficiency: 1.326
Slope: -8.160
YIntercept: 27.04
Link: 0.0493
-5
-4
-3
-2
-1
Log Concentration

Figure S2. Gonadosomatic indices and gonad mass

Morphs do not differ in absolute gonad mass, but when gonad mass is corrected by body mass to create a gonadosomatic index [GSI = (gonad mass x 100) / body mass], Faeders have larger relative testes size compared to Satellites and Independents. Asterisks indicate statistically clear differences between morphs, * p<0.05, *** p<0.001

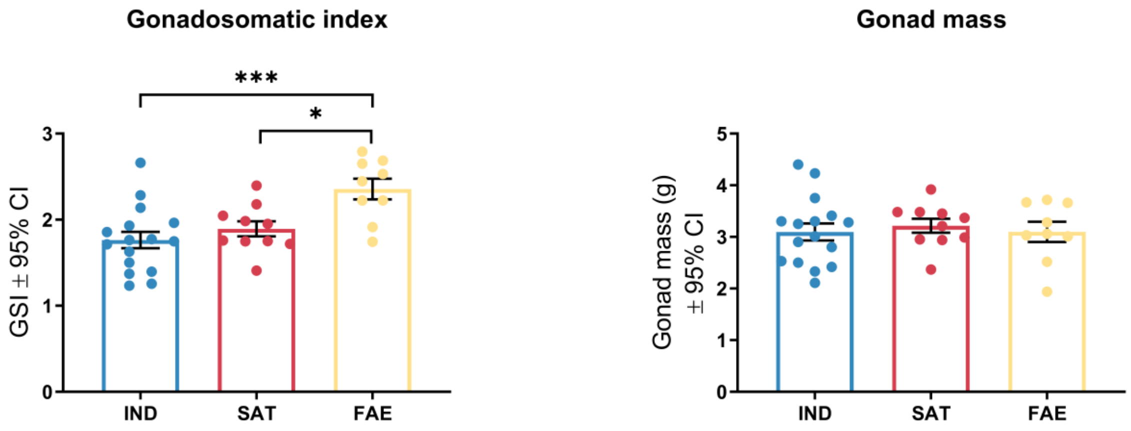

Figure S3. LDA analysis with sequential removal of genes with heaviest loadings

Panels A-D are shown in the main manuscript in Fig. 5. Panels E-I depict subsequent linear discriminant analyses that we performed by removing the heaviest positive and negative LD1 loadings following the table below. The dataset size refers to the number of genes. The separation of Faeders from the other two morphs largely persisted even after both inversion genes (*HSD17B2* and *SDR42E1*) were removed, as evidence even in panel H. All three morphs clustered together only in the three-gene dataset (I).

Panel	Dataset size	Genes removed
A	14	None
B	13	*ESR2*
C	13	*HSD17B2*
D	12	*ESR2, HSD17B2*
E	10	*LHR, PGR*
F	8	*ESR1, SRD5A1*
G	6	*SDR42E1, SRD5A2*
H	4	*FSHR, AR*
I	3	*STAR*

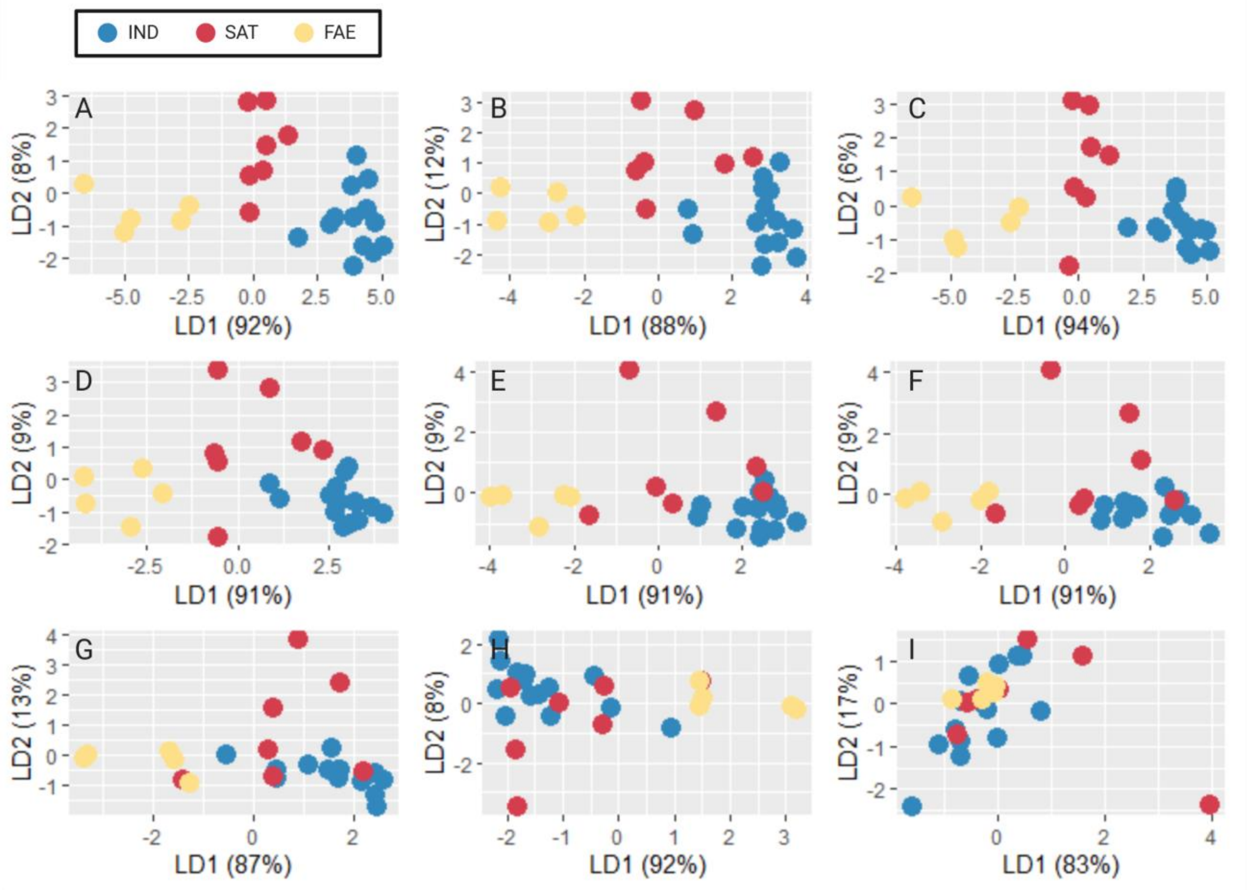

Figure S4. LDA analysis with sequential removal of genes with lightest loadings

Panel A depicts the LDA analysis with the full 14 gene dataset (same as Fig. 5A). Panels B-G depict subsequent linear discriminant analyses that we performed by removing the lightest positive and negative LD1 loadings following the table below. The dataset size refers to the number of genes. The separation of Faeders from the other two morphs largely persisted up until the 8 gene dataset (D). In the following panels, still some separation of Independents from inversion morphs is visible, but all three morphs also overlap (E, F). Morphs finally cluster in (G).

Panel	Dataset size	Genes removed
A	14	None
B	12	*ARO, HSD17B3*
C	10	*SRD5A2, HSD3B2*
D	8	*AR, SDR42E1*
E	6	*STAR,ESR1*
F	4	*LHR, HSD17B2*
G	2	*FSHR, SRD5A1*

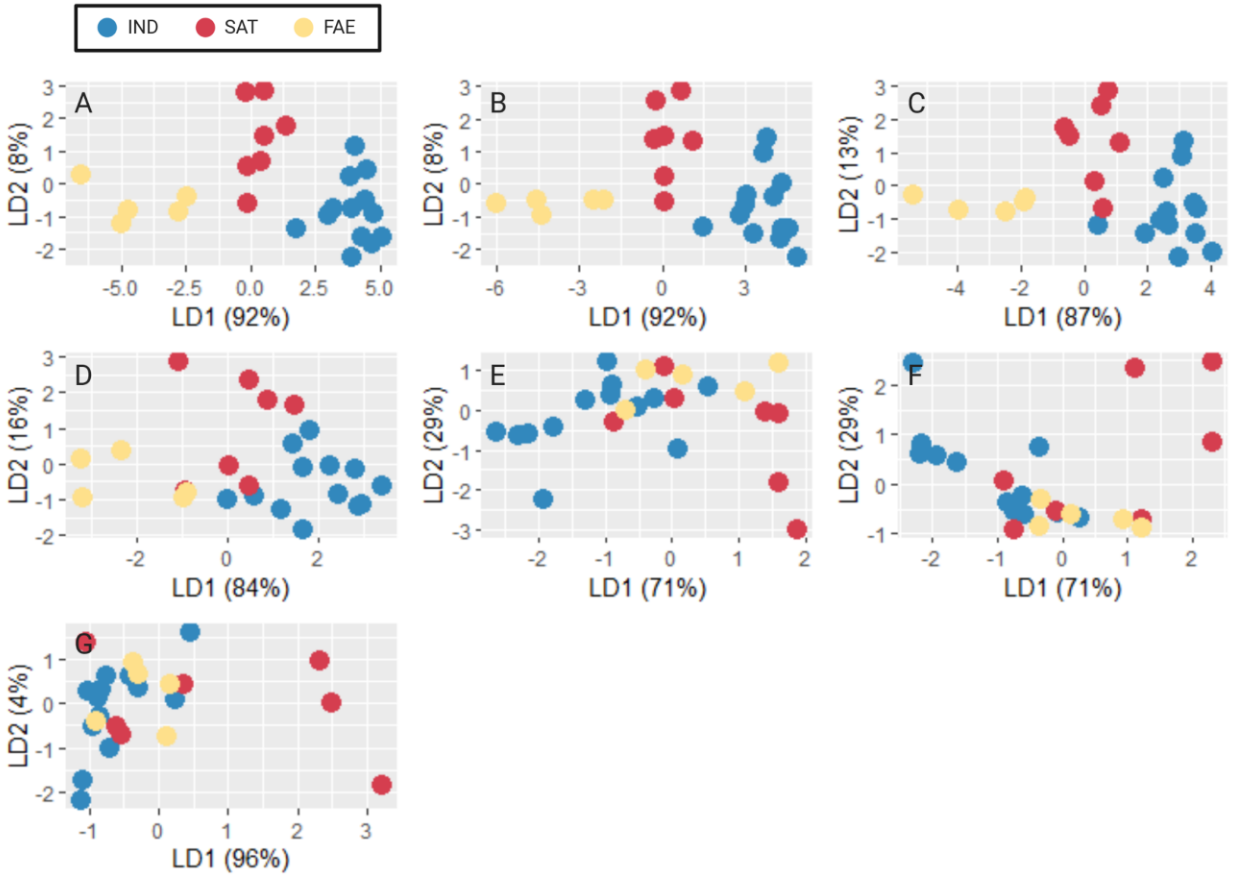

Supplement: Supplementary file 1 [file Data_Sheet_1.pdf]
