# Supplementary material for: Gene Expression Modification by an Autosomal Inversion Associated With Three Male Mating Morphs
Source: Front Genet. 2021 Jun 4;12:641620. doi: 10.3389/fgene.2021.641620 (PMC8213371; doi:10.3389/fgene.2021.641620)
Supplement: Supplementary file 2 [file Data_Sheet_2.docx]

# Supplementary Tables

# Table S1: Primer details

Gene names, accession numbers, primer sequences and information on allele-specific primers are listed. Previously validated genes and primers are listed in Loveland et al. (2021). The ‘Intended allele annealed’ column indicates the haplotype where the primer is intended to anneal. 'Anc' is the ancestrally ordered (non-inverted) haplotype associated with the Independent morph; 'S/F' refers to the inversion haplotype in a region where Satellite and Faeder share the same SNP; ‘F’ Faeder allele; 'Anc/S/F' indicates there is no SNP that distinguishes any of the morphs and therefore the primer anneals to both non-inverted (ancestrally ordered) and inversion haplotypes. For the *SPATA2L* gene, positions where allele specific primers differed besides the last base on the 3' end are shown in boldface. For the *ZDHHC7* gene, the sequence 'GCG' was added to the 5' end of each reverse primer to increase their respective melting temperatures.

| **Gene** | **Accession** | **Type** | **Primer** | **Sequence 5'-3'** | **Intended allele annealed** |
| --- | --- | --- | --- | --- | --- |
| *BCO1* | [XM_014942394.1](https://www.ncbi.nlm.nih.gov/nuccore/XM_014942394.1?from=193&to=1773&sat=4&sat_key=153522502&report=fasta) | Inversion | bco1.F.Anc | GCTTGGCTCTGCTGCACAC | Anc/S/F |
|  |  |  |  |  |  |
|  |  |  | bco1.REV | GACACCACGATTCGGTTTGC | Anc/S/F |
| *CENPN* | [XM_014942117.1](https://www.ncbi.nlm.nih.gov/nuccore/XM_014942117.1) | Inversion | CENPN.out.F | CTGAGGCACAGCTGAAAACC | Anc/S/F |
|  |  |  | CENPN.out.R | AGGACAGCTGCTTGCTTTATG | Anc/S/F |
|  |  |  | CENPN.across.F1 | CGACTTCGGTCAGCCTTGAA | Anc |
|  |  |  | CENPN.across.R1 | GTATACGGTGTTCCCCAGGC | Anc |
| *HSD17B2* | [XM_014942225.1](https://www.ncbi.nlm.nih.gov/nucleotide/960965828?report=genbank&log$=nucltop&blast_rank=1&RID=UXK4TK8V01R) | Inversion | hsd17b2.F.Anc | GGAAGGTGCTGATCCGGAGCC | Anc/S/F |
|  |  |  | hsd17b2.REV | CCCAGTGTCGCTTCCTGTGAT | Anc/S/F |
| *PLCG2* | [XM_014942591.1](https://www.ncbi.nlm.nih.gov/nuccore/XM_014942591.1?from=44&to=3841&sat=4&sat_key=153522704&report=fasta) | Inversion | plcg2.F.SH3 | GCTTCTCTCGAGGAGCTTTAAT | Anc/S |
|  |  |  | plcg2.R.SH3 | CCGTAATCTCCCTTCCACCAG | Anc/S |
|  |  |  | plcg2qF1 | GGAAACCAGGCAGGTAGCAT | Anc/S/F |
|  |  |  | plcg2qR1 | TTTGCTTTGCAACGCTCGAA | Anc/S/F |
| *SCL7A5* | [XM_014942373.1](https://www.ncbi.nlm.nih.gov/nuccore/XM_014942373.1?from=78&to=1673&sat=4&sat_key=153522482&report=fasta) | Inversion | scl7a5qF1 | CTACCTGCTCAAGCCCATCT | Anc/S/F |
|  |  |  | scl7a5qR1 | ACACGAGTGGCTGCTTTCAC | Anc/S/F |
| *SDR42E1* | [XM_014942222.1](https://www.ncbi.nlm.nih.gov/nucleotide/960965823?report=genbank&log$=nucltop&blast_rank=1&RID=UXK1X2RS01R) | Inversion | sdr42e1.F.Anc | GTCAAGTCTGGTTTATACAAGCACG | Anc/S/F |
|  |  |  | sdr42e1.REV | AGCTACAGATTTGGTCCGGG | Anc/S/F |
| *SPATA2L* | [XM_014942220.1](https://www.ncbi.nlm.nih.gov/nuccore/XM_014942220.1?from=167&to=1366&sat=4&sat_key=153522321&report=fasta) | Inversion | spata2L.F.Anc | ACACGAGGCGAGAGGACAT | Anc/S/F |
|  |  |  | spata2L.F.Inv | ACA**T**GAGGC**A**AGAGGACAC | S/F |
|  |  |  | spata2L.REV | TCCAGGTTGGGGTTGCTTG | Anc/S/F |
| *TERF2* | [XM_014942519.1](https://www.ncbi.nlm.nih.gov/nuccore/XM_014942519.1?from=11&to=1570&sat=4&sat_key=153522636&report=fasta) | Inversion | terf2.F.Anc | CTACTTCTACCAGGCCATGG | Anc/S/F |
|  |  |  | terf2.REV | AATCGAGGTTCTCGCCTTCC | Anc/S/F |
| *ZDHHC7* | [XM_014942452.1](https://www.ncbi.nlm.nih.gov/nuccore/XM_014942452.1?from=1&to=957&report=fasta) | Inversion | zdhhc7.F1 | GTCTTTGGGGGTCAGCCTTC | Anc/S/F |
|  |  |  | zdhhc7.R.Faed | GCGTCAAACAGAAAACTCA | F |
|  |  |  | zdhhc7.R.IndSat | GCGTCAAACAGAAAACTCG | Anc/S |
| *ZFPM1* | [XM_014942579.1](https://www.ncbi.nlm.nih.gov/nuccore/XM_014942579.1?from=74&to=3481&sat=4&sat_key=153522690&report=fasta) | Inversion | zfpm1.F.Faed | AGTGCACCGCCTGCCA | F |
|  |  |  | zfpm1.F.IndSat | AGTGCACCGCCTGCCG | Anc/S |
|  |  |  | zfpm1.REV | CCCTTCATCTTCTGGAGGTGCTC | Anc/S/F |
| *ZNF469* | [XM_014942127.1](https://www.ncbi.nlm.nih.gov/nuccore/XM_014942127.1?from=1&to=12486&sat=4&sat_key=153522223&report=fasta) | Inversion | zinf469.F.Faed | AAAAGCTCTTGTCAGCAGAGAC | F |
|  |  |  | zinf469.F.IndSat | AAAAGCTCTTGTCAGCAGAGAG | Anc/S |
|  |  |  | zinf469.REV | GGCTTTGCTTCTGGAATCGC | Anc/S/F |
| *HSD17B3* | [XM_014936139.1](https://www.ncbi.nlm.nih.gov/nuccore/XM_014936139.1?from=77&to=1009&report=fasta) | Outside inversion | 17b3_F635 | CTGGGTACTTTCCCTTGCCC | NA |
|  |  |  | 17b3_R765 | CCATAAGGAGCCACTACCTGT | NA |
| *HSD3B2* | [XM_014957124.1](https://www.ncbi.nlm.nih.gov/nuccore/XM_014957124.1?from=210&to=1343&report=fasta) | Outside inversion | 3b2_F335 | TAGCAATGAAGCCCTCGGGA | NA |
|  |  |  | 3b2_R477 | CAATGATGGAAGCCGTGTGG | NA |
| *SRD5A1* | [XM_014940764.1](https://www.ncbi.nlm.nih.gov/nuccore/XM_014940764.1?from=1&to=963&report=fasta) | Outside inversion | SDR5a1_F536 | TGCTAGCGCTTCTGTTCTGT | NA |
|  |  |  | SDR5a1_R675 | GATTGCCATGCCGACTAACC | NA |
| *SRD5A2* | [XM_014950809.1](https://www.ncbi.nlm.nih.gov/nuccore/XM_014950809.1?from=7&to=774&report=fasta) | Outside inversion | SDR5a2_F425 | ATTGGTGCACCGACATCAGA | NA |
|  |  |  | SDR5a2_R541 | AAGTGACTTCCCCGGGTTTC | NA |

# Table S2. Single nucleotide polymorphisms (SNPs) in inversion genes tested for allelic imbalance

We tested four inversion genes for allelic imbalance and designed primers based on SNPs that were either unique to Faeders or shared between Satellites and Faeders. SNP position information is listed for each gene along with its corresponding amino acid in the ancestral allele and the inversion allele(s) in Satellites and Faeders.

Abbreviations: ‘*Inv*’, the inversion haplotype (associated with either Satellite or Faeder); ‘*Anc*’, the ancestrally ordered haplotype; ‘Syn’, synonymous substitution; ‘Non-syn’, non-synonymous substitution, ‘IND’, Independent; ‘SAT’, Satellite, ‘FAE’, Faeder.

|  | | | | | | | | | | |
| --- | --- | --- | --- | --- | --- | --- | --- | --- | --- | --- |
| Gene |  | IND | SAT | FAE | SNP | | Amino acid | | |  |
|  |  | *+/+* | *+/Inv* | *+/Inv* | Position^a^ | Type | IND | SAT | FAE |  |
| *SPATA2L* |  | TT | TC | TC | 695 | Non-syn | I | **T** | **T** |  |
| [*ZDHHC7*](https://www.ncbi.nlm.nih.gov/nuccore/XM_014942452.1?from=1&to=957&report=fasta) |  | CC | CC | CT | 942 | Syn | P | P | P |  |
| *ZFPM1* |  | GG | GG | GA | 2705 | Non-syn | R | R | **H** |  |
| *ZNF469* |  | GG | GG | GC | 1817 | Non-syn | S | S | **T** |  |
|  |  |  |  |  |  |  |  |  |  |  |
| ^a^ SNP position is based on the Genbank coding sequence. Gene accession numbers are listed in Table S1. | | | | | | | | | | |

**Table S3. Primer efficiencies for non-inversion genes**

| **Gene** | **Efficiency** | **Slope** | **Error** | **Y-intercept** |
| --- | --- | --- | --- | --- |
| *HSD17B3* | 1.950 | -3.447 | 0.026 | 19.800 |
|  |  |  |  |  |
| *HSD3B2* | 1.994 | -3.337 | 0.033 | 19.120 |
|  |  |  |  |  |
| *SRD5A1* | 2.040 | -3.227 | 0.056 | 25.380 |
|  |  |  |  |  |
| *SRD5A2* | 1.971 | -3.394 | 0.035 | 23.910 |

# Table S4: One-sample t-test results for allelic imbalance in inversion morphs

For each gene and tissue combination we performed a one-sample t-test against a hypothetical mean of 50%, with the contribution from the inversion allele expressed as a percentage of total (biallelic) expression. Unadjusted p-values are shown below the test-statistic. To account for multiple testing we applied a Bonferroni-Dunn correction and those that retained significance are shown in bold.

|  | *ZFPM1* | *ZDHCC7* | *ZNF469* | *SPATA2L* |
| --- | --- | --- | --- | --- |
| Adrenal glands | t_1_=23.67 | t_1_=8.41 | t_1_=4.91 | t_3_=9.71 |
|  | 0.027 | 0.075 | 0.128 | **0.002** |
| Liver | t_1_=3.08 | t_1_=4.68 | t_1_=1.00 | t_3_=8.51 |
|  | 0.2 | 0.134 | 0.5 | **0.003** |
| Gonads | t_1_=3.81 | t_1_=8.59 | t_1_=0.62 | t_3_=4.43 |
|  | 0.163 | 0.074 | 0.648 | 0.021 |
